# Assessment of Lifetime Risk for Cardiovascular Disease: Time to Move Forward

**DOI:** 10.2174/011573403X311031240703080650

**Published:** 2024-07-03

**Authors:** Evangelia G. Sigala, Demosthenes B. Panagiotakos

**Affiliations:** 1 Department of Nutrition and Dietetics, School of Health Sciences and Education, Harokopio University of Athens, 70 El. Venizelou, Kallithea, 176 76, Athens, Greece

**Keywords:** Lifetime risk, cardiovascular disease, risk assessment, epidemiology, harmonization, hypercholesterolemia

## Abstract

Over the past decades, there has been a notable increase in the risk of Cardiovascular Disease (CVD), even among younger individuals. Policymakers and the health community have revised CVD prevention programs to include younger people in order to take these new circumstances into account. A variety of CVD risk assessment tools have been developed in the past years with the aim of identifying potential CVD candidates at the population level; however, they can hardly discriminate against younger individuals at high risk of CVD.Therefore, in addition to the traditional 10-year CVD risk assessment, lifetime CVD risk assessment has recently been recommended by the American Heart Association/American College of Cardiology and the European Society of Cardiology prevention guidelines, particularly for young individuals. Methodologically, the benefits of these lifetime prediction models are the incorporation of left truncation observed in survival curves and the risk of competing events which are not considered equivalent in the common survival analysis. Thus, lifetime risk data are easily understandable and can be utilized as a risk communication tool for Public Health surveillance. However, given the peculiarities behind these estimates, structural harmonization should be conducted in order to create a sex-, race-specific tool that is sensitive to accurately identifying individuals who are at high risk of CVD. In this review manuscript, we present the most commonly used lifetime CVD risk tools, elucidate several methodological and critical points, their limitations, and the rationale behind their integration into everyday clinical practice.

## 1 INTRODUCTION

Despite the decline in Cardiovascular Disease (CVD) mortality and the almost negligible decreases in incident cases over the past decades, prevalence has remained unchanged or has even risen in some countries, while CVD-related deaths account for one-third of all-cause premature mortality (*i.e.,* death before the age of 70), establishing CVD as the principal contributor to disability-adjusted life years [1]. Simultaneously, upward trends have been observed in morbidity indices of predisposing risk factors, which in turn further exacerbate the disease burden [1, 2]. More than ever before, CVD events occur at an unprecedented incidence among younger individuals, even before the age of 40 [2, 3]. These trends impose immense strains on public health resources and exert a profound detrimental effect on individuals' quality of life [1]. Hence, the primordial and primary prevention of CVDs has been acknowledged as a priority within the scientific community [4-7] and by policymakers [8, 9].

To reinforce prevention actions, risk prediction models have been developed with the aim of identifying high-risk individuals for CVD events early in everyday clinical practice [10-12]. Most of these tools are employed to prognosticate the occurrence of fatal, non-fatal, or combined events over a predefined and relatively short time frame, typically set at 10 years. Among others, examples of total CVD risk algorithms include the pioneer Framingham Risk Score (FRS) [13], the Pooled Cohort Equations (PCE) [5, 7, 14], the Systematic Coronary Risk Evaluation (SCORE) [4, 15-18], the QRISK scores [19-21], the MESA score [22, 23], the ASSIGN score [24, 25], the Reynolds Risk Scores [26, 27], the PROCAM risk score [28], and the INTERHEART Modifiable Risk Score (IHMRS) [29]. Evidence suggests that utilizing a score depicting the absolute risk within a fixed and brief time window as a motivational technique has been proven to be ineffective [30], particularly when applied to younger persons whose calculated absolute short-term CVD risk is mostly low [10-12, 30, 31] –albeit it is often underrated– with the exception of those with monogenic disorders, such as homozygous familial hypercholesterolemia [11]. Therefore, recent guidelines by multi-national scientific societies advocate for the incorporation of lifetime risk assessment for CVD in their prevention strategies [4-6]. A marked benefit of this approach is that it provides a more accurate representation of real-life risks, particularly for apparently healthy, younger individuals. Moreover, it is more readily understandable for both individuals [30] and healthcare professionals [32], especially when compared to relative risk estimates [33]. Thus, lifetime risk scores may serve as valuable tools for effectively communicating the significance of promptly initiating or intensifying interventions, including lifestyle modifications.

There are few studies that provide an overview of lifetime risk in the context of CVD epidemiology, despite the fact that multiple authors [10-12, 34-37] have critically reviewed the convectional CVD risk prediction tools. Therefore, this study aims to present the most commonly used lifetime CVD risk tools, elucidate several methodological and critical points raised, including limitations, and underscore the rationale behind their integration into everyday clinical practice.

## 2 THE CONVENTIONAL CVD RISK ESTIMATION TOOLS

Before introducing lifetime CVD risk models, the conventional CVD risk estimation tools are briefly described. The statistical notion of CVD risk prediction appears to have originated from a mid-1950s US landmark study, the Framingham Heart Study. This study not only introduced the term “risk factor” [38], but also pioneered the development of the first scoring calculator for assessing coronary heart disease (CHD) risk in adults, known as the Framingham Risk Score (FRS) [13]. Since then, numerous risk assessment tools have emerged, designed with the principal objective of identifying potential candidates who would experience a positive net clinical benefit when initiating or intensifying CVD risk-reducing interventions [10, 12]. Extending the FRS, contemporary risk estimation systems go beyond focusing solely on CHD or other single-outcome risk approaches, as these events share the same risk factors. Instead, these models accommodate additional and multiple cause-specific endpoints, like stroke, to derive a composite score that estimates the total or global CVD risk [11]. These outcomes are well-defined hard endpoints encompassing fatal and non-fatal events [13]. This enhances the reliability of the calculators, *e.g.*, throughout recalibration processes, as these available data are based on more robust and unbiased assessments [11].

A shared feature of these CVD risk scores is the computation of the absolute risk of experiencing fatal and/ or non-fatal atherothrombotic events over a specified time horizon, usually 10 years, after accounting for a range of predictors. Estimating the absolute risk provides several advantages, including enabling the evaluation of the trade-offs between the potential benefits and adverse effects of available preventive interventions, as well as facilitating risk communication in high-risk groups [12], since it seems to be easier to understand [39]. For example, the calculation of absolute risk emphasizes that individuals with a higher pretreatment risk have the potential to obtain greater benefits from CVD preventive measures compared to those with lower risks, notwithstanding the fact that they may receive an equivalent relative risk reduction from a particular treatment [31]. In Table **1**, an overview of the most commonly used CVD risk scores, detailing their main strengths and weaknesses, is presented. Apart from the well-recognized limitations of these prediction models, which have been extensively documented in prior reviews [10-12, 34-37], a notable drawback of the most conventional CVD risk prediction equations employing standard survival analysis is the absence of competing event adjustments, *i.e*., events that prevent the occurrence of the endpoint of interest (Table **1**). Instead, these risk scores treat competing events as censored observations, implying that non-CVD endpoints cannot exist, violating the assumption of independent censoring [40-42]. Similarly, the assumption of non-informative censoring is compromised, as this approach implies that subjects who developed a non-CVD event can be represented by those who have not yet experienced any outcome and remain under follow-up.

Another remarkable weakness of CVD risk scores that has received less attention is that age is often treated solely as a non-modifiable risk factor in the majority of the models (Table **1**), examining its significance as “exposure duration” to various CVD predisposing risk factors [10, 11, 43]. Therefore, the relationship of age with clinical and other risk factors is not static; instead, this dynamic relationship should be incorporated into the risk models as time-varying effects [44]. Additionally, to address the dynamic effect of aging and its interactions with socio-demographic determinants, lifestyle choices, and clinical conditions, even in a crude approach, it is imperative to conduct an age-specific analysis of the risk [10]. Nevertheless, age-stratified analyses employed in CVD risk scores are applicable only within certain age ranges, typically starting from middle age and reaching up to 70-79 years old, depending on the characteristics of the baseline cohorts [10, 12]. Moreover, younger adults are expected to be labeled as having “low-” absolute CVD risk in a short-term time interval of 5-10 years [10-12, 31]. However, a substantial subset of these individuals may genuinely have increased CVD risk in the long term while maintaining the same unfavorable heart-healthy behaviors [11]. Hence, this group may exhibit an increased relative risk compared to those who have adopted a healthy lifestyle, making them potential candidates for early preventive measures such as lifestyle modification.

Several approaches have been suggested to communicate the calculated CVD risk to adults. These techniques include recalibrating the perceived CVD risk, which almost always encompasses an optimistic bias [45, 46]; estimating the relative risk, *i.e.*, the risk compared with a person of the same sex and age but with optimal risk factor levels [45]; prognosticating the estimated life expectancy [47]; and calculating the risk age, the heart age, and the lifetime risk [14, 45, 47, 48]. Recently, the European Society of Cardiology [4] and American Heart Association / American College of Cardiology [5, 6] guidelines have highlighted the importance of lifetime risk estimation, especially for asymptomatic younger people with low absolute risk. In the next section, a detailed presentation of the concept of lifetime risk in CVD epidemiology will follow.

## 3 THE CONCEPT OF LIFETIME RISK

Lifetime risk reflects the cumulative probability of developing a medical condition, such as a disease, a specific event, or a risk factor, over an individual’s expected lifespan [57, 58]. Therefore, lifetime risk is mathematically modeled as a cumulative incidence, utilizing age as the principal time scale [59]. Assuming that an advanced age point represents the upper boundary of the estimated lifespan, the time origin of a lifespan can be defined either from birth [60] or from a specified age time-point, referred to as the “age index” [58, 59], at which individuals are free of the health condition. Usually, at this age index, the population-based risk rises significantly, and thus, it would be beneficial to further investigate. When an age index is considered, the “residual or remaining lifetime risk” is calculated, reflecting the cumulative incidence of a health condition among individuals who have reached the age index, without experiencing the condition. It should be mentioned that since left truncation is inherent in these models, some subjects may have developed the condition prior to their enrollment in the study [61]. Nevertheless, these individuals are excluded with the aim of ensuring that all sampled persons have comparable time origins [62].

### 3.1 Competing Risks and Lifetime Risk Modelling

Given that the competing risk of CVD increases with age, lifetime risk models should be adjusted for competing events [59], which should not be treated as censored data [41]. Competing risks arise when modeling non-terminal events or cause-specific mortality [63]. For instance, if CVD-related mortality is the event of interest, deaths due to non-CVD causes should be treated as a competing outcome and not as censored observations in the analysis; notwithstanding, the latter is an equivalent outcome of survival. Thus, in that case, there would be a risk of experiencing the event of interest, *i.e*., CVD death, after non-CVD death [58]. A meta-analytic pooled analysis of 77 studies suggested that not considering competing outcomes, as in the classic univariate Kaplan-Meier method, may result in an overestimation of the cumulative incidence of a specific condition [64]. Similar biased findings were observed in a recently published study focusing on secondary CVD prevention, in which unadjusted Cox models were compared to equations incorporating competing events [65]. Clinically, this impedes the cost-effectiveness of the scores, as it suggests that a greater number of patients would be referred to receive therapy when the unadjusted models are employed. Hence, accommodating competing risk adjustments in models is essential, especially when aiming for accurate risk prognoses, particularly in high-risk populations.

Several studies estimating CVD-related lifetime risk [33, 57, 66-68] have employed a non-parametric analysis, specifically a modified Kaplan-Meier method, in which competing risks of non-CVD deaths are considered and age represents the time scale [57, 58, 62]. Supposing that competing events are absent, we define T as a random variable associated with the survival times of individuals and the sequence. 
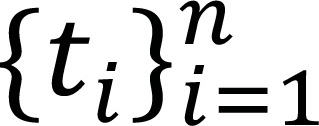
 represents the ordered time values of events among b_0_ = N individuals, where n < N, who are initially free of the outcome of interest at t_0_ [57, 58, 62, 69]. Then, the number of subjects who are at risk of experiencing the event of interest beyond t_i_, *i.e.,* the risk set, is b_i_. According to the potential outcomes, we define:

d_i_: the number of subjects who experience new events of interest at time t_i_;

c_i_: the number of persons who are censored at time t_i_.

The traditional Kaplan-Meier (KM) estimator of survival beyond t_i_ can be computed as described in Table **2**:


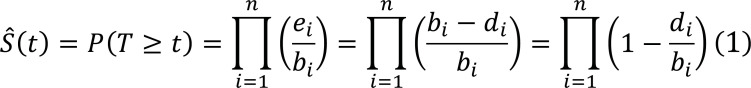


The hazard function, denoted as h(t) or λ(t), is equal to the ratio d_i_/b_i_ and represents the instantaneous rate of experiencing the event of interest at time t_i_, given that participants have survived until t_i-1_ [69]. Thus, it can be expressed by the formula:


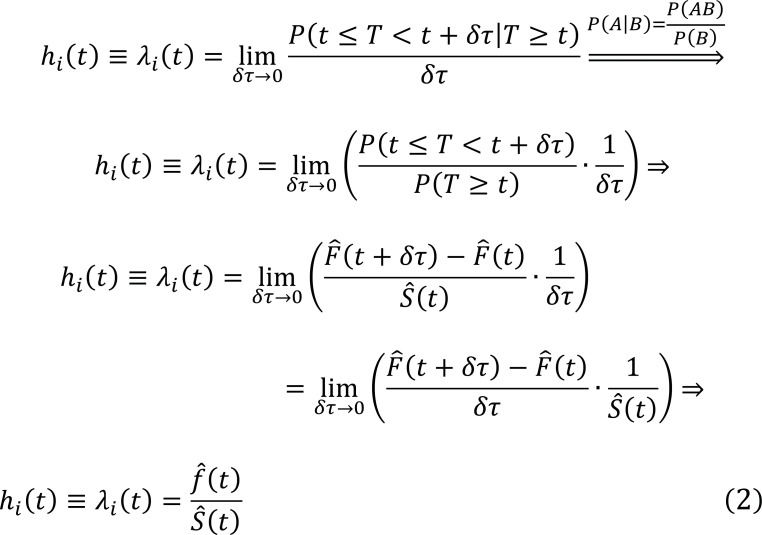


Where 
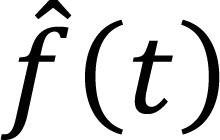
 is the probability density function, whose integration gives the cumulative incidence function, 
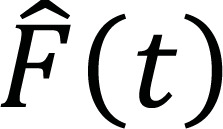
, which for discrete-time events can be expressed as the sum:


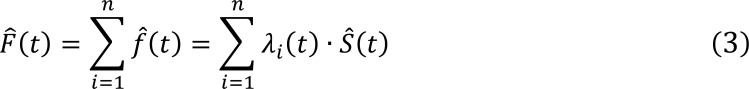


and since 
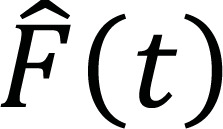
 is the complement of the KM estimator, the survival function can be written as:


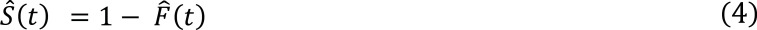


Assuming that the time scale expresses age, a, then the hazard of experiencing the event at age a is h_a_, which is assumed to be zero for ages less than an a_min_, which is the lowest age threshold at which an event can occur [62]. Then, the remaining or residual lifetime risk can be defined as the Cumulative Incidence Function (CIF) up to an upper limit of age, a_max_:


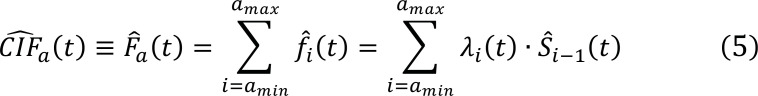


With the variance calculated utilizing Greenwood’s formula:


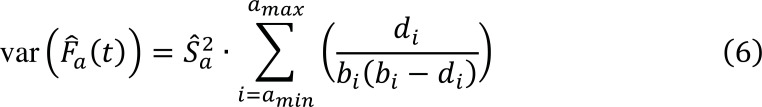


Therefore, a 95% confidence interval (CI) can be expressed in the space:


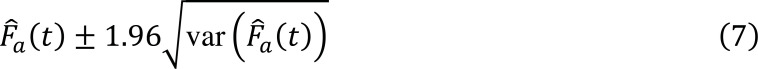


This analysis has not been adjusted for competing events.

To adjust for competing events, we define g_i_ as the number of persons who experience an incident event of interest and a competing event at age a_i_, thus d_i_ + r_i_ (Table **3**). The corresponding hazard is equal to g_i_/b_i_ at age a_i_. Then, the survival probability is defined as:


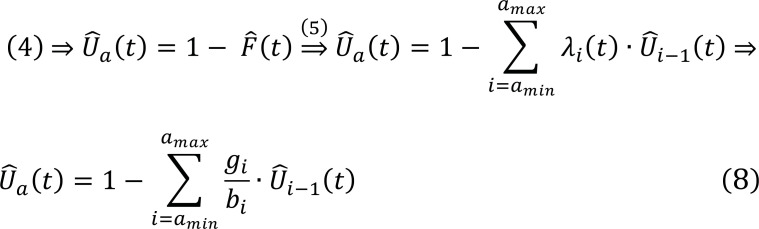


Then, the correspondent probability density function can be expressed as:


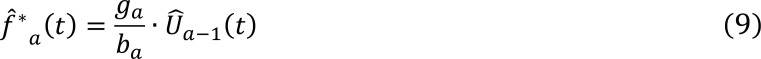


Considering Eqs. (3) and (9), the remaining or residual lifetime risk-adjusted for competing risks is written by the formula:


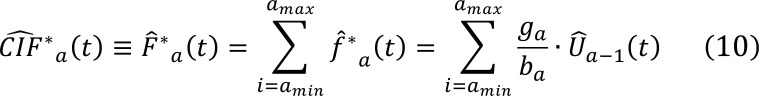


In other studies that have conducted competing risk analysis, two commonly presented hazard functions are the cause-specific hazard function 
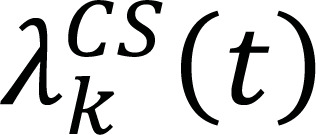
, and the Fine-Gray subdistribution hazard function 
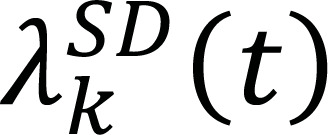
, [42]. For the k^th^ event, these functions are defined as follows:


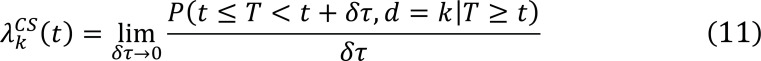


In the first case, the cause-specific CIF, or the remaining or residual lifetime risk for the k^th^ event, can be expressed as:


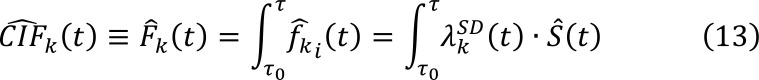


For the Fine-Fray model, we assumed that 
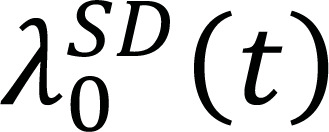
 is the baseline subdistribution hazard, **X_i_** are the explanatory variables, and **b** are the regression coefficients [59]. To account for left truncation, we define:


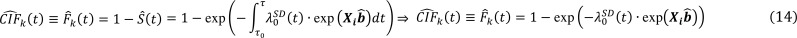


For more details regarding the mathematical modeling of lifetime risk, readers are referred to the publication of Conner *et al.* [59].

### 4 USE OF LIFETIME RISK AS A TOOL FOR PRIMARY CVD PREVENTION

In contrast to most risk scores that employ a fixed short-term time window, lifetime risk equations intrinsically incorporate advancing age as an increasing exposure duration in risk factors [70]. Seshadri and Wolf [58] recognized two patterns of lifetime risk estimates depending on the pathophysiological pathways of the underlying condition that drive its trajectories. In the first pattern, the lifetime risk of experiencing a certain event may be increased in younger individuals, but as the remaining disease-free life expectancy declines and the probability of experiencing other competing events rises, the lifetime risk of developing this event subsequently decreases or flattens afterward [58, 71]. This approach adequately reflects a real-world illustration of the dynamic lifetime trajectories of nutritional-related non-communicable diseases (NCD), which are particularly prevalent in countries that have shifted to the later stages of nutritional transition, and, thus, the burden of NCD is significant. The second pattern is applicable to age-related diseases; as age advances, lifetime risk rises in line with age-specific incidence rates. Almost precisely, this increase compensates for the reduction in remaining life expectancy [58].

Lifetime risk estimates have been previously published for a range of health conditions, including cancer [72-75] (*e.g.*, breast [60, 76, 77], lung [78], skin [79], colon [80], ovaries [81], endometrium [82], prostate [83]), neurological diseases (*e.g.*, Alzheimer Disease [62, 84, 85] and total dementias [86]), mental and psychiatric disorders [87, 88], autoimmune diseases [89], HIV [90], diabetes mellitus [91, 92], chronic kidney disease [93] and end-stage kidney disease [94, 95], osteoporotic fractures [96, 97], systematic [98] and pulmonary [99] hypertension, and impaired glucose metabolism [100]. Regarding CVDs, the existing literature has focused on either a composite score of CVDs [33, 67, 68, 101] or cause-specific cardiac outcomes, such as coronary heart disease [57, 102-104], stroke [58, 105-108], and atrial fibrillation [39, 109-113].

Considering that lifetime risks genuinely represent long-term mortality-adjusted absolute risk estimates [33, 58], which are easily interpreted, these estimates can be incorporated in clinician-patient discussion [114], in order to facilitate risk communication and effectively encourage behavioral changes towards adopting beneficial lifestyle habits [39, 115, 116]. Nevertheless, it is imperative to complement lifetime risk estimates with short-term risk assessment, because the former corresponds to population-based estimates that do not discriminate between the potential of short-term or long-term events in the future [58]. On the other hand, at an individual level, additional factors, such as risk profile and management, as well as comorbidities, should also be taken into account.

At a societal macro-level, lifetime risk estimates serve as a valuable tool for Public Health surveillance. Specifically, this statistic allows the long-term evaluation of the disease burden in a population, the prediction of future disease trajectories, and realistic comparisons between known diseases in a population [33]. Hence, these estimates could guide policymakers in the strategic allocation of financial and human resources to strengthen health systems, as well as in advocating for public health initiatives that promote early screening and heart-healthy lifestyles, such as educational and awareness-raising campaigns. For example, one of the most widely cited lifetime risk statistics is that of breast cancer. According to this estimate, 1 in 8 women at birth in the USA will develop breast cancer over their life course [60]. This statistic was used by the American Cancer Society to raise awareness about breast cancer among women [58]. Nowadays, lifetime risk assessment has been integrated into breast cancer primary prevention guidelines, which recommend a score of 20% as the cut-off point defining the “at-risk” category [117]. Therefore, women with a lifetime risk score higher than this threshold are recommended to undergo breast magnetic resonance imaging at an annual rate, along with mammography. Examples of lifetime risk tools for breast cancer include the Tyrer-Cuzick model [117] and the Gail model [118-121]. Similarly, the long and insidious nature of atherosclerotic processes [35], the rising trends in incident CVDs among younger individuals over the past decades [2, 3], the fact that clinical risk factors may not have clinically manifested yet in this age-group, as well as the ambiguity regarding the risk factor treatment thresholds in these individuals [122], along with the fact that the 10-year CVD algorithms underestimate CVD risk in apparently healthy younger adults with established risk factors for CVDs [11, 12], have extended the concept of lifetime risk in the research field of CVDs, as a tool to detect potential CVD candidates [11, 12] promptly and to promote the adoption of healthy lifestyle habits [123]. Therefore, lifetime risk assessment has been integrated into multi-national evidence-based guidelines for CVD prevention.

The 2013 ACC/AHA Guidelines on the Assessment of CVD Risk [14] and the Blood Cholesterol Guidelines [123] were the first official guidelines to incorporate the lifetime risk assessment as an adjunctive tool to the 10-year CVD risk calculators in daily clinical practice. Specifically, despite the weak level of evidence (Grade C), it was suggested that all atherosclerotic CVD-free adults aged 20 to 39 years, as well as individuals between 40 and 59 years old with a 10-year risk of less than 7.5% (low-to-borderline risk), should be screened using either a 30-year or the lifetime risk for a first atherosclerotic CVD (ASCVD) event, which encompasses traditional risk factors, including age, sex, total cholesterol, HDL-C cholesterol, systolic blood pressure (BP), BP-lowering agents, presence of diabetes mellitus (DM), and smoking status [14, 123]. A web-based calculator was also suggested. The primary role of lifetime risk assessment would be for risk communication purposes, in particular, to motivate potential future CVD candidates to adopt behavioral modification for a life-long, heart-healthy lifestyle, while it was highlighted that there was no evidence supporting its use in making decisions regarding pharmacotherapy [14]. Additionally, although this assessment might not provide additional value for those who are already identified at high-risk thresholds using the conventional 10-year calculators, it could still serve as a useful tool for motivation, aiming to increase adherence to lifestyle changes and drug therapy. The updated 2018 Guidelines for Management of Blood Cholesterol [5] and the 2019 ACC/AHA Guideline on the Primary Prevention of CVD [6] maintained these recommendations, emphasizing the significance of incorporating a 30-year or a lifetime ASCVD risk assessment for directing clinician-patient discussions towards the intensity of lifestyle modification. Similarly, the 2016 Guidelines on CVD Prevention of the European Society of Cardiology (ESC) suggested a lifetime risk tool, such as the online JBS3 calculator, due to its potential to identify high-risk individuals, even when their 10-year risk score is low, both in the short and long term [124]. Nevertheless, these guidelines also highlighted the research gaps regarding its usefulness in critical appraisal of decision-making related to prescribed pharmacotherapy. In the subsequent 2021 ESC Guidelines, a two-step approach for CVD risk assessment is suggested [4]. The first step involves prevention goals for all individuals, irrespective of their risk level, and the next step entails risk stratification and intensification of prevention and treatment. In both steps, for apparently healthy adults (*i.e.*, individuals without established ASCVD, DM, chronic kidney disease (CKD), and genetic lipid or BP disorder) in the high-risk CVD risk category, as assessed by SCORE2 (calculated score between 2.5% and 7.5% for persons under the age of 50 and between 5% and 10% for individuals aged between 50 and 69 years), lifetime risk, lifetime treatment benefit, risk modifiers, polypharmacy, and patient preferences should be considered. It was also acknowledged that lifetime risk might be a more useful communication tool for younger individuals, whose 10-year risk is consistently low. The LIFEtime-perspective Cardiovascular Disease (LIFE-CVD) was suggested as a calculator for lifetime risk assessment. Recently, this model has been updated to the LIFE-CVD2 model, which incorporates regional differences in CVD risk across Europe [125].

### 4.1 Tools for Lifetime CVD Risk Estimation

In Table **4**, algorithms that estimate lifetime risk are presented, including the Lifetime ASCVD Risk Plus, which is the extension of the 10-year PCE over the lifespan [14], the QRISK-lifetime (QLifetime) score, the LIFE-CVD [126] and the LIEF-CVD2 [125], the IBERLIFERISK score [127], and the IBERLIFERISK2 score [128]. Beyond the aforementioned methodological advantages of lifetime risk models over the convectional CVD risk models with fixed short-term time horizon, another important advantage is that the majority of these tools are available as user-friendly applications (apps) or online calculators (Table **4**).

Beginning with the methodological drawbacks that have also been highlighted in the short-term CVD risk estimation systems, a methodological weakness in the ASCVD Risk Estimator Plus is its reliance on older cohorts characterized by higher CVD morbidity and mortality rates [10]. Specifically, PCE was based on cohorts’ baseline examinations conducted approximately 30-50 years ago. Thus, when extrapolated to contemporary populations, the estimates may tend to overestimate the risk. Another source of misestimation in all calculators, except for LIFE-CVD2 [125], is the inclusion of risk-modifying predictors as stable over time and not as time-varying covariates, *i.e.*, assuming that exposure to certain levels of risk factors remains stable over the years and basing calculations solely on baseline data collected approximately 20-30 years ago (Table **4**). This approach fails to accommodate temporal changes, thereby potentially introducing biases into CVD risk estimation. Additionally, although these cohorts encompass mostly large, gender-balanced samples, the age of the sampled individuals during the recruitment of the samples resulted in different final age ranges of the models (Table **4**). Also, the samples have diverse socio-cultural, geographical, genetic, and behavioral attributes. For instance, the PCE is modeled on data from non-Hispanic whites and African Americans, restricting their application to populations with multiple ethnicities. Furthermore, the applicability of these tools can be compromised in individuals with underlying chronic inflammatory diseases, such as rheumatoid arthritis [12]. Therefore, considering these peculiarities, along with the dynamic interactions between risk factors and environmental conditions, as well as the existence of unknown risk factors that have not been accounted for yet in the equations, these models may have limited comparability and predictive ability in different populations with different characteristics [10]. Thus, recalibrating lifetime risk models based on contemporary and representative region-specific epidemiological data of CVD events and relative risk factors, as in LIFE-CVD2, is suggested to enhance the predictive ability of the models [125].

Moreover, different sets of risk-modifying predictors and statistical methods have been applied to these lifetime risk models. For instance, in IBERLIFERISK2, apart from the traditional risk factors, body mass index (BMI) and alcohol consumption have also been added (Table **4**). In the PCE model, the competing events have not been take into account. Similarly, these calculators do not consider the same endpoints, such as heart failure, which is only considered in IBERLIFERISK2. Therefore, the applicability of these scores in populations other than the baseline cohort and the generalization of the results could potentially result in risk misestimation. For example, in a recent study, the short-term and the lifetime PCE had different predictive abilities compared to the correspondent QRISK calculators, which was attributed to the inherent differences in the characteristics of the models, including the age ranges, predictors, and statistical models [129].

Another important weakness inherent to lifetime risk models is their method of validation, due to the long-term time frame of prediction that often extends beyond the study duration [130]. However, the models may be calibrated using a diagram depicting the estimated *versus* observed lifetime risks while taking into consideration the presence of competitive events over a 5- or 10-year follow-up per risk decile [128]. Last but not least, lifetime risk may be subject to birth cohort effects [33, 130, 131].

### 4.2 Use of Lifetime CVD Risk to Support Epidemiological Evidence

Several epidemiological studies have published lifetime risk estimates for CVDs and their cause-specific events. Focusing on studies that calculated estimates based on a global CVD score rather than each type of event separately, numerous studies have shown that many individuals are categorized as “low risk” when risk assessment tools with short-term time windows are employed; notwithstanding, many of them are identified as high risk based on a lifetime risk assessment [140, 141]. Notably, findings from CARDIA indicated that subjects in the latter category (*i.e.,* low short-term risk/ high lifetime risk) had greater subclinical disease burden, as assessed by common and internal carotid intima-media thickness, as well as by coronary artery calcium (CAC) [142]. Supporting evidence from the Dallas Heart Study suggests that among individuals with low short-term risk and CAC = 0, those with high lifetime risk have a greater incidence of calcified plaques in the coronary arteries at follow-up [143].

One important study was that of Lloyd-Jones *et al.,* which examined the Framingham Heart Study cohort's lifetime risk for CVD by risk factor burden at age 50. [33]. It was found that at 50 years of age, men had a 51.7% (95% CI: 49.3–54.2%) probability of developing CVD at any time during their remaining lifespan, with a median survival of 30 years. The corresponding risk for women was much lower, at 39.2% (95% CI: 37.0–41.4%), with median survival of 36 years. Additionally, it was found that individuals with more than 2 major risk factors (including smoking status, diabetes, low HDL-C, as well as elevated total cholesterol (TC), BP, and BMI) had a significantly higher lifetime risk of experiencing a CVD event and much lower median survivals (men: 68.9% (95% CI: 61.7–73.2%), women: 50.2% (95% CI: 44.7–55.7%); median survival in men: 28 years and women: 31 years), compared to those who had favorable risk factor profile (men: 5.2% (95% CI: 0–12.2%), women: 8.2% (95% CI: 0–22.3%); median survival in men and women: > 39 years). Similar results were found in the Chicago Heart Association Detection Project in Industry [144]. Specifically, middle-aged adults, between 40 and 59 years old, with at least 3 risk factors (*i.e*., untreated hypertension, hypercholesterolemia, smoking, BMI ≥ 25 kg/m^2^) had greater residual lifetime risk for CVD (men: 35.2% (95% CI: 31.5–38.8%), women: 31.9% (95% CI: 27.6–36.2%)) and shorter survival (median survival in men: 26 years and women: 28 years), in comparison with those who had optimal levels of risk factors (men: 20.5% (95% CI: 11.6–29.4%), women: 6.7% (95% CI: 2.2–11.1%); median survival in men and women: >35 years). These findings agree with the results of other longitudinal studies [144, 145], as well as the finding of a meta-analysis that unveiled that an optimal risk factor burden in middle age is associated with a reduced risk of mortality up to 80 years of age and a lower lifetime risk of fatal or non-fatal CVD events (CHD, MI, stroke) [131].

In Greece, until recently, the lifetime risk for CVD had never been presented. However, in a recent publication by Panagiotakos *et al.* [68], lifetime risk was predicted based on the data from the ATTICA epidemiological Study. It was reported that the long-term, *i.e*., 20-year, crude CVD incidence for men ranged from 7.4% at the age of 40 years to 89.0% at the age of 60 years, and for women, it ranged from 2.8% to 90.1% at the ages of 40 and 60 years, respectively. Moreover, the lifetime risk for fatal and non-fatal CVD events was 68% (95% CI: 64–73%) for men and 63% (95% CI: 60–65%) for women at the index age of 40 years old, and as age free of CVD increased, lifetime risk for CVD events decreased, and lifetime risk estimates showed a progressive decline from 68% (95% CI: 64–73%) to 55% (95% CI: 51– 59%) and from 63% (95% CI: 60–65%) to 55% (95% CI: 46–64%), at the index ages of 40 to 60 years, in men and women, respectively. Women had a similar lifetime CVD risk as compared to men at all index ages (*p*-value = 0.245). This temporal pattern of lifetime risk coincides with the first pattern described by Seshadri and Wolf [58] that reflects the underlying pathophysiology.

## 5 TIME TO MOVE FORWARD: ESTIMATING LIFETIME RISK FOR CVD PREVENTION

Globally, policymakers and health organizations acknowledge that CVDs are the outcome of the interplay of multiple risk factors, some of which are modifiable [146, 147]. The management of these modifiable risk factors through national and international policies and targets has been the main focus of efforts to address CVD. .European policies support reducing clinical risk factors and promoting improvements in lifestyle factors like food, exercise, alcohol use, and smoking [147]. Similarly, the WHO has integrated CVD prevention in the Global Action Plan for the prevention and control of NCD [148], while the United States has prioritized NCD prevention in the 3.4 Targets of the Sustainable Development Goals [9, 91].

CVD risk assessment is an intimate means for identifying potential candidates who would receive a positive net clinical benefit from early-initiating preventive interventions [10, 12]. However, short-term CVD risk tools fail to detect many individuals with high lifetime risk [140, 141]. Therefore, the lifetime risk assessment should be integrated into preventive CVD screening for all young people. Besides, these estimates could be used as motivation not only for young people but also for older people with established risk factors who need motivation for intensified behavioral change towards a beneficial lifestyle habit and adhering to pharmacotherapy. At this point, it should be mentioned that telemedicine, including m-Health, e-Health, telemonitoring, and telehealth [149], provides a high potential for primary CVD prevention in high-risk patients [150]. Results from the LIGHT randomized clinical trial have indicated that m-Health interventions effectively improve clinical and lifestyle-related risk factors, along with biomarkers predisposing to CVD. Furthermore, the utilization of telemedicine has been associated with reduced unnecessary outpatient services and patient transfers, lower in-hospital mortality rates, improved compliance with medication, and enhanced quality of life [149].

However, considering that lifetime risk estimates are population-based cumulative estimates that cannot predict the timing of an event, such as pre-mature CVD incidents, these tools should either be adjutant to short-term calculators [58] or incorporate additional risk factors that, at an individual level, are important and may be precursors of subclinical atherosclerosis and other CVD pathology. Indeed, according to a recent study by the Global Cardiovascular Risk Consortium, harmonized individual-level data from more than 1.5 million participants worldwide was indicative of 57.2% and 52.6% of incident cases of CVDs among women and men, respectively [151, 152]. Similarly, given the peculiarities between the cohorts in which lifetime risk estimates have been calculated, structural harmonization should be employed to reduce these variations. Thereafter, clinically important factors that contribute to the early prediction of CVD outcomes, such as lifestyle factors and social determinants, should be considered with the ultimate objective of creating a lifetime risk calculator that could be predictive of CVD outcomes irrespective of regional parameters, such as the European LIFE-CVD2. Additionally, lifetime risk models could be extended to encompass younger age brackets and associated with lifestyle indices, such as the MediLIFE-index [153], to promote lifestyle modifications even at early ages, thereby enhancing the effective primordial prevention of CVD.

Future studies should investigate the role of lifetime risk as a risk communication tool for all individuals. Considering that the incorporation of a lifetime risk assessment would result in higher rates of adults diagnosed as high-risk, higher rates of adults would be directed toward therapy initiating for longer periods. Therefore, the cost-effectiveness of this approach to the health systems should be examined. Additionally, considering the long insidious period of atherosclerosis, lifetime risk assessment should be examined in children with clinical risk factors for CVD. Moreover, in light of the widely acknowledged role of telemedicine, forthcoming guidelines should incorporate lifetime risk assessment into tele-consultations.

## CONCLUSION

Lifetime risk can be utilized to communicate potential CVD hazards, inspire modifications in lifestyle habits, or even enhance compliance with pharmacological treatment. Moreover, lifetime risk estimates can potentially guide Public Health surveillance and provide insights regarding the allocation of future resources, as they can be used for the long-term assessment of the known diseases’ burden in a population and the prediction of future disease trajectories.

## AUTHORS’ CONTRIBUTIONS

It is hereby acknowledged that all authors have accepted responsibility for the manuscript's content and consented to its submission. They have meticulously reviewed all results and unanimously approved the final version of the manuscript.

## Figures and Tables

**Table 1:** Conventional models estimate CVD risk within a fixed and relatively short time horizon.

**CVD Risk Tool (Acronym) [REF.]**	**Cohorts’ Characteristics**	**Predictors**	**Endpoints**	**Strengths**	**Limitations**
Framingham Risk Score (FRS) [13]	Prospective studies, enrolling the general population in Framingham, USA. 8,491 CVD-free individuals, 46.7% men, 30-74 years.	Age, sex, TC, HDL-C, DM, SBP, antihypertensive treatment, smoking. Sex-specific score.	10-year risk of CVD events (CHD, stroke, PAD, HF).	Longitudinal data. Not limited to CHD as [49]. Hard endpoints.	Mainly Caucasians, thus limited applicability in populations with other racial background. No competing risks.
Pooled Cohort Equations (PCE) [14]	Prospective studies, enrolling the general population in the USA. 24,238 CVD-free individuals, 42.7% men, 40-79 years.	Age, sex, race, TC, HDL-C, DM, SBP, antihypertensive treatment, smoking. Sex- and race-specific scores (non-Hispanic white men and women, non-Hispanic African American men and women).	10-year risk of CVD events (CHD death, non-fatal MI, non-fatal stroke, stroke death).	Longitudinal data. Better representation of non-Hispanic African Americans than FRS. Hard endpoints. Available online [50, 51].	Mainly Caucasians and African Americans, thus limited applicability in populations with other racial background. No competing risks.
Systematic Coronary Risk Evaluation (SCORE [15] and SCORE2 [16])	Prospective studies from European countries, including population-based and occupational cohorts. SCORE: 205,178 CVD-free individuals, 57.1% men, 40-65 years. SCORE2: 677,684 CVD-free individuals, 44.4% men, 40-79 years (applicable to age range: 40-69 years).	Age, sex, TC or TC: HDL-C ratio, SBP, smoking. Sex- and region-specific analyses, based on the risk profile of countries.	SCORE: 10-year risk of CVD mortality (CHD, PAD, ischaemic stroke). SCORE2: 10-year risk of fatal (hypertensive disease, IHD, HF, stroke, atherosclerosis, sudden death) and non-fatal (non-fatal MI, non-fatal stroke) CVD events.	Longitudinal data from several European cohorts. Region-specific risks. In SCORE2, age interaction terms added as predictors and models adjusted for competing events. Available online [52].	Only fatal events included in SCORE, underestimating the total CVD risk. Limited applicability in populations beyond Europe.
Systematic Coronary Risk Evaluation – Older Persons (SCORE-OP [18] and SCORE2-OP [17])	Prospective studies from European countries. SCORE-OP: 40,825 CHD-free individuals, 50.7% men, ≥65 years. SCORE2-OP: 367,098 CVD-free individuals, 42.5% men, ≥65 years (applicable to ≥70 years).	Age, sex, TC, HDL-C, DM, SBP, smoking. Sex- and region-specific analyses, based on the risk profile of countries.	SCORE-OP: 10-year risk of CVD mortality (CHD, PAD, ischaemic stroke). SCORE2-OP: 5- and 10-year risk of fatal (CHD, HF, sudden death) and non-fatal (non-fatal MI, non-fatal stroke) CVD events.	Longitudinal data from several European cohorts. Region-specific risks. In SCORE2, age interaction terms added as predictors and models adjusted for competing events. Available online [52].	Only fatal events included in SCORE-OP, underestimating the total CVD risk. Limited applicability in populations beyond Europe.
The QRISK1 [21], QRISK2 [19], and QRISK3 [20] scores	QRESEARCH electronic database of health records of general practices in England and Wales, UK. QRISK1: 1.28 million CVD-free individuals, 49.6% men, 35-74 years. QRISK2: 1.54 million CVD-free individuals, 49.6% men, 35-74 years. QRISK3: 7.89 million CVD-free individuals, 59.7% men, 25-84 years.	QRISK1: Age, sex, TC:HDL ratio, SBP, antihypertensive treatment, history of CHD in 1^st^ degree relative aged < 60, smoking, deprivation, BMI. QRISK2, as in QRISK1, along with ethnicity and the presence of chronic diseases. QRISK3, as in QRISK2, includes SBP variability, CKD G3-5, migraine, corticosteroid use, SLE, atypical antipsychotic medications, severe mental illness, HIV/AIDs, and erectile dysfunction. Sex-specific scores.	10-year risk of CVD events (MI, CHD, stroke, TIA).	Incorporation of additional risk factors, such as BMI, social deprivation, chronic nosological conditions, and other routinely collected data, as well as interaction effect terms. Large sample. Lack of selection and participation biases. In QRISK2 and QRISK3, age interaction terms added as predictors. Available online [53].	Prospective open cohort design resulting in relative short median follow-up. Substantial imputation of missing values. Ethnicity was self-assigned. False positive diagnoses. No competing risks.
The MESA score [22, 23]	The Multi-ethnic Study of Atherosclerosis (MESA), enrolling prospectively general population in the USA. 6,814 CVD-free individuals, 47.2% men, 45-85 years.	Age, sex, ethnicity, TC, HDL-C, lipid-lowering medication, DM, SBP, antihypertensive treatment, CAC, family history of heart attack, smoking. Sex- and race-specific scores (White, Black, Hispanic, and Chinese).	10 years of CVD events (MI, resuscitated cardiac arrest, fatal CHD, fatal and non-fatal stroke).	Longitudinal data. Multi-ethic cohort, including Whites, Blacks, Hispanics, and Chinese. Age interaction terms and CAC added as predictors. Available online [54].	No competing risks.
The ASSIGN score Version 1.5.1 [24, 25]	Prospective study, enrolling the general population in Scotland. 13,297 CVD-free individuals, 49.2% men, 30-74 years.	Age, sex, TC, HDL-C, DM, SBP, family history of CHD or stroke, social deprivation, smoking, RA. Sex-specific scores.	10-year risk of fatal and non-fatal (CHD, coronary artery interventions, stroke) CVD events.	Longitudinal data. Tailored for the Scottish population. Incorporation of additional risk factors, such as family history, social deprivation. Available online [25].	Limited applicability in populations beyond Scotland. No competing risks.
The Reynolds Risk Score [26, 27]	RCTs, enrolling women and men healthcare providers. 35,282 CVD-free individuals, 30.4% men, men: ≥50 years old, women: ≥45 years old.	Age, sex, TC, HDL-C, Apo-B-100, Lp(a) (if Apo-B-100 ≥ 100), HbA1c (if DM), hs-CRP, SBP, parental history of MI < 60 years old. Sex-specific score.	10-year risk of MI, stroke, coronary revascularization, and CVD death.	Incorporation of additional risk factors, such as family history and hsCRP, as well as interaction terms. Available online [55].	Mainly Caucasians, thus limited applicability in populations with other racial background. Gender-biased score. No competing risks.
The PROCAM risk score [28]	The Prospective Cardiovascular Münster study, enrolling prospectively employees in Germany. 26975 individuals, 68.4% men, 20-75 years.	Age, sex, LDL-C, HDL-C, TG, DM, SBP, family history of MI, smoking. Sex-specific scores.	10-year risk of major coronary event (sudden death, fatal or non-fatal MI, cardiac enzyme changes) or 10-year risk of stroke (ischemic stroke, TIA).	Longitudinal data. Tailored for the German population. Available online [56].	Limited applicability in populations beyond Germany. Gender-biased score. Not global CVD events. No competing risks.
The INTERHEART Modifiable Risk Score (IHMRS) [29]	The case-control study was conducted in 52 countries in Asia, Europe, the Middle East, Africa, Australia, North America, and South America. 19470 individuals, 75.3% men, 20-75 years.	Age, Apo-B:A1 ratio, DM, SBP, WRH, smoking, second-hand smoking. Other variables accounted for in precedent models: psychosocial factors (stress, depression, perceived locus of control, adverse life events), dietary factors (consumption of food group), physical activity, and parental history of MI at any age.	Acute MI.	Large international study, with a multi-ethnic sample. Incorporation of modifiable risk factors and age interaction terms.	Case-control design. Only MI, not global CVD.

**Abbreviations:** Apo-B-100: apolipoprotein B-100, Apo-B:A1 ratio: ratio of apolipoprotein B to apolipoprotein A1, BMI: body mass index, CAC: coronary artery calcification, CHD: coronary heart disease, CKD G3-5: chronic kidney disease stages 3-5, CVD: cardiovascular disease, DM: diabetes mellitus, ESC: european society of cardiology, HDL-C: high-density lipoprotein-cholesterol, HF: heart failure, hsCRP: high-sensitivity C-reactive protein, IHD: ischemic heart disease, Lp(a): lipoprotein (a), LDL-C: low-density lipoprotein-cholesterol, MI: myocardial infraction, PAD: peripheral arterial disease, RA: rheumatoid arthritis, REF: references, RCT: randomized control trial, SBP: systolic blood pressure, SLE: systemic lupus erythematosus, TC: total cholesterol, TG: triglycerides, TIA: transient ischemic attack, WHR: waist-to-hip ratio.

**Table 2:** The Kaplan-Meier life table.

**Time (t_i_)**	**Risk Set (b_i_)**	**No. of Events (d_i_)**	**No. of Censorings (c_i_)**	**No. of Subjects Survived (e_i_)**	**Survival Function (S(t_i_))**
t_0_	b_0_	d_0 _= 0	c_0_ = 0	e_0_ = b_0_	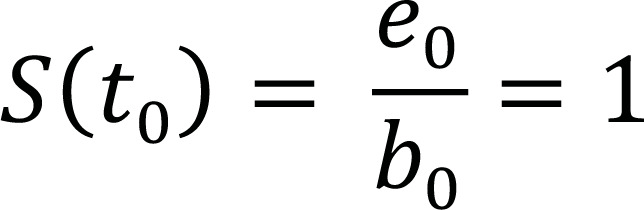
t_1_	b_1_ = e_0_	d_1_	c_1_	e_1_ = b_1_ – (d_1_ + c_1_)	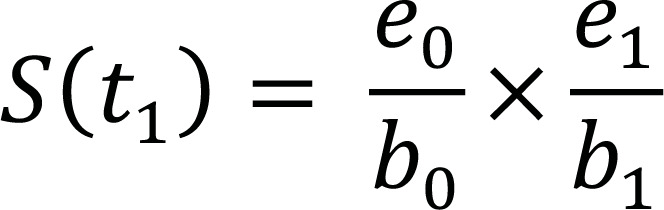
t_2_	b_2_ = e_1_	d_2_	c_2_	e_2_ = b_2_ – (d_2_ + c_2_)	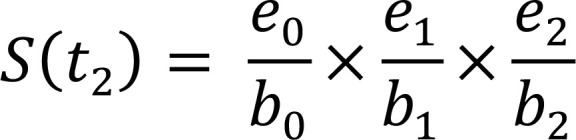
⁝	⁝	⁝	⁝	⁝	⁝
t_n_	b_n_ = e_n-1_	d_n_	c_n_	e_n_ = b_n_ – (d_n_ + c_n_)	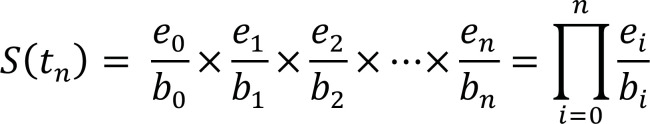

**Table 3:** The Kaplan-Meier life table accounting for competing events.

**Age (a_i_)**	**Risk Set (b_i_)**	**No. of Events (d_i_)**	**No. of Comp. Events (r_i_)**	**No. of Subjects Survived (e_i_)**	**Survival Function (U(t_i_))**
a_min_	b_0_	d_0 _= 0	r_0 _= 0	e_0_ = b_0_	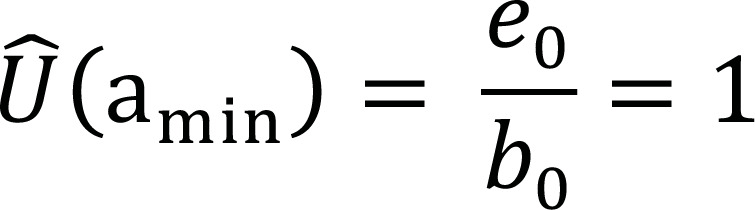
a_1_	b_1_ = e_0_	d_1_	r_1_	e_1_ = b_1_ – (d_1_ + r_1_)	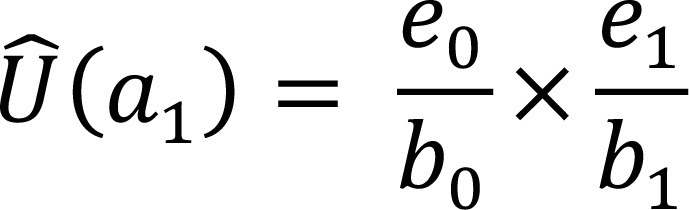
a_2_	b_2_ = e_1_	d_2_	r_2_	e_2_ = b_2_ – (d_2_ + r_2_)	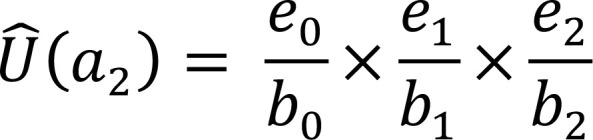
⁝	⁝	⁝	⁝	⁝	⁝
a_max_	b_n_ = e_n-1_	d_n_	r_n_	e_n_ = b_n_ – (d_n_ + r_n_)	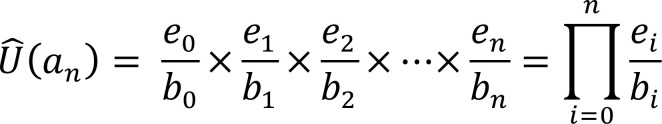

**Table 4:** Lifetime risk models.

**CVD Risk Tool (Acronym) [REF.] **	**Cohorts’ Characteristics**	**Predictors **	**Endpoints**	**Statistical Methodology**	**Calibration, Discrimination, and Validation **
ASCVD Risk Estimator Plus [5, 33] (Pooled Cohort Equations, PCE) Available online [50].	Prospective studies, enrolling the general population in the USA. >30,000 CVD-free individuals, 20-59 years.	Age, sex, race, TC, HDL-C, LDL-C, lipid-lowering treatment, DM, SBP, DBP, antihypertensive treatment, aspirin, smoking. Sex- and race-specific scores (non-Hispanic white men and women, non-Hispanic African American men and women).	Lifetime risk of CVD events (CHD death, non-fatal MI, non-fatal stroke, stroke death).	A modified Kaplan-Meier.	-
The QRISK3-lifetime (QLifetime) score [132] (recommended by JBS3 [133] and NICE [134] guidelines) Available online [135].	Research electronic database of health records of general practices in England and Wales, UK. Data collected from 1994–2010 from GP databases – imputation of missing data. 126,716 CVD-free individuals, 73.7% men, 25-84 years.	Age, sex, ethnicity, CKD, DM, AF, SBP, antihypertensive treatment, migraines, RA, SLE, mental illness, antipsychotic meditation, steroid tablets, erectile dysfunction, smoking, TV: HDL-C ratio, height, weight. Sex-specific scores.	Lifetime risk of CVD events (MI, CHD, stroke, TIA).	Cox proportional hazard models with cause-specific hazards.	External validated with primary care, hospital, and mortality data [136]. Internal validation with 1,27 million patients: Of the 10% of patients in the validation cohort classified at highest risk with either the lifetime risk model or the 10 year risk model, only 14.5% were at high risk on both measures.
Lifetime-perspective CardioVascular Disease (LIFE-CVD) [114], its updated version [137] Available online [138].	Prospective studies encompassing North American and European cohorts. 6,715 CVD-free individuals, 53% men, 45-80 years.	Age, sex, ethnicity, non-HDL-C, DM, SBP, parental history of MI prior to age 60, BMI, smoking. Sex- and race-specific scores (White, Black, Hispanic, and Chinese).	Lifetime risk of fatal or non-fatal CVD (MI, stroke, resuscitated cardiac arrest, CHD-event, death). Additional outcomes include a 10-year risk, CVD-free life expectancy, and a lifetime treatment effect.	Fine and Gray models.	External validated with 1,451,077 CVD-free individuals [139]. Calibration: Harrell’s C-indices: from 0.670 (95% CI: 0.650–0.690) to 0.787 (95% CI: 0.785–0.789) [139].
Lifetime-perspective CardioVascular Disease2 (LIFE-CVD2) [125].	Prospective studies in 13 countries encompassing North American and European cohorts. 687,135 CVD-free individuals, 43% men, 35-100 years.	Age, sex, TC, HDL-C, DM, SBP, smoking. Sex-, age- and region-specific scores.	Lifetime risk of fatal or non-fatal CVD (MI, stroke, death). Additional outcome: lifetime treatment benefit.	Cox proportional hazard models with cause-specific hazards.	External validated with 1,657,707 CVD-free individuals from 8 European cohorts. Discrimination: Harrell’s C-index of 0.795 (95% CI: 0.767–0.822). Calibrated in population-wide e-health records data in the UK and Netherlands.
The IBERLIFERISK [127] and the IBERLIFERISK2 [128] Available online [140]	Retrospective Spanish occupational cohort. Baseline: 2004-2007. IBERLIFERISK: 762,054 CVD-free individuals, 71.1% men, 18 -65 years. IBERLIFERISK2: 762,058 CVD-free individuals, 53% men, 18-75 years.	Age, sex, TC, lipid-lowering treatment, CKD, DM, SBP, DBP, antihypertensive treatment, history of CVD in 1^st^-degree relative, smoking, occupation, alcohol consumption, BMI.	Lifetime risk of fatal or non-fatal CVD (CHD, HF, cerebrovascular diseases, PAD, death due to hypertensive disease, death due to arrhythmia).	Cox proportional hazard models with cause-specific hazards. IBERLIFERISK2: up to 2014, IBERLIFERISK2: up to 2017.	Calibration: IBERLIFERISK: Underestimation in low-risk deciles and overestimation in high-risk deciles. IBERLIFERISK2: A slight degree of underestimation in women and overestimation in men in the last decile of risk Spiegelhalter’s Z: not statistically significant in both sexes. Discrimination: IBERLIFERISK: AUC: 0.84 (95% CI: 0.82–0.85) in men and 0.73 (95% CI: 0.66–0.80) in women. IBERLIFERISK2: Harrell’s C-index: 0.78 (95% CI: 0.76–0.79) in men and 0.73 (95%: CI 0.69–0.77) in women AUC: 0.82 for the men and 0.73 for the women.

**Abbreviations:** AF: atrial fibrillation, AUC: area under the curve, BMI: body mass index, CHD: coronary heart disease, CKD: chronic kidney disease, CVD: cardiovascular disease, DBP: diastolic blood pressure DM: diabetes mellitus, HDL-C: high-density lipoprotein-cholesterol, HF: heart failure, LDL-C: low-density lipoprotein-cholesterol, MI: myocardial infraction, PAD: peripheral arterial disease, RA: rheumatoid arthritis, REF: references, SBP: systolic blood pressure, SLE: systematic lupus erythematosus, TC: total cholesterol, TIA: transient ischemic attack.

## LIST OF ABBREVIATIONS

CHD: Coronary Heart Disease
CVD: Cardiovascular Disease
FRS: Framingham Risk Score
FRS: Framingham Risk Score
PCE: Pooled Cohort Equations
TC: Total Cholesterol

## References

[1] Timmis A., Vardas P., Townsend N. (2022). European Society of Cardiology: cardiovascular disease statistics 2021.. Eur. Heart J..

[2] Roth G.A., Mensah G.A., Johnson C.O. (2020). Global Burden of Cardiovascular Diseases and Risk Factors, 1990–2019.. J. Am. Coll. Cardiol..

[3] Sun J., Qiao Y., Zhao M., Magnussen C.G., Xi B. (2023). Global, regional, and national burden of cardiovascular diseases in youths and young adults aged 15–39 years in 204 countries/territories, 1990–2019: a systematic analysis of Global Burden of Disease Study 2019.. BMC Med..

[4] Visseren F.L.J., Mach F., Smulders Y.M. (2021). 2021 ESC Guidelines on cardiovascular disease prevention in clinical practice.. Eur. Heart J..

[5] Grundy S.M., Stone N.J., Bailey A.L. (2019). 2018 AHA/ACC/AACVPR/AAPA/ABC/ACPM/ADA/AGS/APhA/ASPC/NLA/PCNA Guideline on the Management of Blood Cholesterol: A Report of the American College of Cardiology/American Heart Association Task Force on Clinical Practice Guidelines.. Circulation.

[6] Arnett D.K., Blumenthal R.S., Albert M.A. (2019). 2019 ACC/AHA Guideline on the Primary Prevention of Cardiovascular Disease: A Report of the American College of Cardiology/American Heart Association Task Force on Clinical Practice Guidelines.. Circulation.

[7] Whelton P.K., Carey R.M., Aronow W.S. (2018). 2017 ACC/AHA/AAPA/ABC/ACPM/AGS/APhA/ASH/ASPC/NMA/PCNA Guideline for the Prevention, Detection, Evaluation, and Management of High Blood Pressure in Adults.. J. Am. Coll. Cardiol..

[8] (2023). SDG target 3.4 reduce by one third premature mortality from non-communicable diseases through prevention and treatment and promote mental health and well-being.. https://www.who.int/data/gho/data/themes/topics/indicator-groups/indicator-group-details/GHO/sdg-target-3.4-noncommunicable-diseases-and-mental-health.

[9] (2016). United States Transforming our world: The 2030 agenda for sustainable development..

[10] Panagiotakos D.B., Stavrinos V. (2006). Methodological issues in cardiovascular epidemiology: the risk of determining absolute risk through statistical models.. Vasc. Health Risk Manag..

[11] Cooney M.T., Dudina A.L., Graham I.M. (2009). Value and limitations of existing scores for the assessment of cardiovascular risk: a review for clinicians.. J. Am. Coll. Cardiol..

[12] Lloyd-Jones D.M., Braun L.T., Ndumele C.E. (2019). Use of risk assessment tools to guide decision-making in the primary prevention of atherosclerotic cardiovascular disease.. J. Am. Coll. Cardiol..

[13] D’Agostino R.B., Vasan R.S., Pencina M.J. (2008). General cardiovascular risk profile for use in primary care: the Framingham Heart Study.. Circulation.

[14] Goff DC, Lloyd-Jones DM, Bennett G (2014). 2013 ACC/AHA guideline on the assessment of cardiovascular risk. Circulation.

[15] Conroy R., Pyörälä K., Fitzgerald A.P. (2003). Estimation of ten-year risk of fatal cardiovascular disease in Europe: the SCORE project.. Eur. Heart J..

[16] Hageman S., Pennells L., Ojeda F. (2021). SCORE2 risk prediction algorithms: new models to estimate 10-year risk of cardiovascular disease in Europe.. Eur. Heart J..

[17] de Vries T.I., Cooney M.T., Selmer R.M. (2021). SCORE2-OP risk prediction algorithms: estimating incident cardiovascular event risk in older persons in four geographical risk regions.. Eur. Heart J..

[18] Cooney M.T., Selmer R., Lindman A. (2016). Cardiovascular risk estimation in older persons: SCORE O.P.. Eur. J. Prev. Cardiol..

[19] Hippisley-Cox J., Coupland C., Vinogradova Y. (2008). Predicting cardiovascular risk in England and Wales: prospective derivation and validation of QRISK2.. BMJ.

[20] Hippisley-Cox J., Coupland C., Brindle P. (2017). Development and validation of QRISK3 risk prediction algorithms to estimate future risk of cardiovascular disease: prospective cohort study.. BMJ.

[21] Hippisley-Cox J., Coupland C., Vinogradova Y., Robson J., May M., Brindle P. (2007). Derivation and validation of QRISK, a new cardiovascular disease risk score for the United Kingdom: prospective open cohort study.. BMJ.

[22] Budoff M.J., Young R., Burke G. (2018). Ten-year association of coronary artery calcium with atherosclerotic cardiovascular disease (ASCVD) events: the multi-ethnic study of atherosclerosis (MESA).. Eur. Heart J..

[23] McClelland R.L., Jorgensen N.W., Budoff M. (2015). 10-Year coronary heart disease risk prediction using coronary artery calcium and traditional risk factors.. J. Am. Coll. Cardiol..

[24] Woodward M., Brindle P., Tunstall-Pedoe H. (2005). Adding social deprivation and family history to cardiovascular risk assessment: the ASSIGN score from the Scottish Heart Health Extended Cohort (SHHEC).. Heart.

[25] (2023). NHS Scotland. ASSIGN Score – prioritising prevention of cardiovascular disease..

[26] Ridker P.M., Paynter N.P., Rifai N., Gaziano J.M., Cook N.R. (2008). C-reactive protein and parental history improve global cardiovascular risk predic-tion: The Reynolds Risk Score for men.. Circulation.

[27] Ridker P.M., Buring J.E., Rifai N., Cook N.R. (2007). Development and validation of improved algorithms for the assessment of global cardiovascular risk in women: the Reynolds Risk Score.. JAMA.

[28] Assmann G., Cullen P., Schulte H. (2002). Simple scoring scheme for calculating the risk of acute coronary events based on the 10-year follow-up of the prospective cardiovascular Münster (PROCAM) study.. Circulation.

[29] McGorrian C., Yusuf S., Islam S. (2011). Estimating modifiable coronary heart disease risk in multiple regions of the world: the INTERHEART Modifiable Risk Score.. Eur. Heart J..

[30] Navar A.M., Wang T.Y., Mi X. (2018). Influence of cardiovascular risk communication tools and presentation formats on patient perceptions and preferences.. JAMA Cardiol..

[31] Jackson R., Lawes C., Bennett D., Milne R., Rodgers A. (2005). Treatment with drugs to lower blood pressure and blood cholesterol based on an individual’s absolute cardiovascular risk.. Lancet.

[32] Gidlow CJ, Ellis NJ, Riley V (2021). Cardiovascular disease risk communication in NHS Health Checks: A qualitative video-stimulated re-call interview study with practitioners. BJGP Open.

[33] Lloyd-Jones D.M., Leip E.P., Larson M.G. (2006). Prediction of lifetime risk for cardiovascular disease by risk factor burden at 50 years of age.. Circulation.

[34] Wilson P.W.F. (2011). Prediction of cardiovascular disease events.. Cardiol. Clin..

[35] Alagona P., Ahmad T.A. (2015). Cardiovascular disease risk assessment and prevention: current guidelines and limitations.. Med. Clin. North Am..

[36] Ruwanpathirana T., Owen A., Reid C.M. (2015). Review on cardiovascular risk prediction.. Cardiovasc. Ther..

[37] Sofogianni A., Stalikas N., Antza C., Tziomalos K. (2022). Cardiovascular risk prediction models and scores in the era of personalized medicine.. J. Pers. Med..

[38] Kannel W.B., McGee D., Gordon T. (1976). A general cardiovascular risk profile: The Framingham study.. Am. J. Cardiol..

[39] Lloyd-Jones D.M., Wang T.J., Leip E.P. (2004). Lifetime risk for development of atrial fibrillation: the Framingham Heart Study.. Circulation.

[40] Putter H., Fiocco M., Geskus R.B. (2007). Tutorial in biostatistics: competing risks and multi‐state models.. Stat. Med..

[41] Huebner M., Wolkewitz M., Enriquez-Sarano M., Schumacher M. (2017). Competing risks need to be considered in survival analysis models for cardiovascular outcomes.. J. Thorac. Cardiovasc. Surg..

[42] Austin P.C., Lee D.S., Fine J.P. (2016). Introduction to the analysis of survival data in the presence of competing risks.. Circulation.

[43] Dhingra R., Vasan R.S. (2012). Age as a risk factor.. Med. Clin. North Am..

[44] Ridker P.M., Cook N. (2005). Should age and time be eliminated from cardiovascular risk prediction models? Rationale for the creation of a new national risk detection program.. Circulation.

[45] Navar A.M., Stone N.J., Martin S.S. (2016). What to say and how to say it.. Curr. Opin. Cardiol..

[46] Petr E.J., Ayers C.R., Pandey A. (2014). Perceived lifetime risk for cardiovascular disease (from the Dallas Heart Study).. Am. J. Cardiol..

[47] Bonner C., Batcup C., Cornell S. (2021). Interventions Using Heart Age for Cardiovascular Disease Risk Communication: Systematic Review of Psychological, Behavioral, and Clinical Effects.. JMIR Cardio.

[48] Karmali K.N., Lloyd-Jones D.M. (2013). Adding a life-course perspective to cardiovascular-risk communication.. Nat. Rev. Cardiol..

[49] Marma A.K., Lloyd-Jones D.M. (2009). Systematic examination of the updated Framingham heart study general cardiovascular risk profile.. Circulation.

[50] (2023). ACC ASCVD Risk Estimator + 2023..

[51] (2023). AHA 2018 Prevention Guidelines Tool CV Risk Calculator..

[52] (2023). EAPC HeartScore..

[53] https://qrisk.org.

[54] (2023). Multi-ethnic study of atherosclerosis. MESA 10-Year CHD risk with coronary artery calcification.. https://www.mesa-nhlbi.org/MESACHDRisk/MesaRiskScore/RiskScore.aspx.

[55] (2023). Reynolds Risk Score. https://www.scymed.com/en/smnxph/phqgg440.htm.

[56] (2017). MDApp. Cardiovascular Risk PROCAM Score Calculator..

[57] Lloyd-Jones D.M., Larson M.G., Beiser A., Levy D. (1999). Lifetime risk of developing coronary heart disease.. Lancet.

[58] Seshadri S., Wolf P.A. (2007). Lifetime risk of stroke and dementia: current concepts, and estimates from the Framingham Study.. Lancet Neurol..

[59] Conner S.C., Beiser A., Benjamin E.J., LaValley M.P., Larson M.G., Trinquart L. (2022). A comparison of statistical methods to predict the residual lifetime risk.. Eur. J. Epidemiol..

[60] Feuer E.J., Wun L.M., Boring C.C., Flanders W.D., Timmel M.J., Tong T. (1993). The lifetime risk of developing breast cancer.. J. Natl. Cancer Inst..

[61] Geskus R.B. (2015). Data analysis with competing risks and intermediate states..

[62] Beiser A., D’Agostino R.B., Seshadri S., Sullivan L.M., Wolf P.A. (2000). Computing estimates of incidence, including lifetime risk: Alzheimer’s disease in the Framingham Study. The Practical Incidence Estimators (PIE) macro.. Stat. Med..

[63] Deo S.V., Deo V., Sundaram V. (2021). Survival analysis—part 3: intermediate events and the importance of competing risks.. Indian Journal of Thoracic and Cardiovascular Surgery.

[64] Lacny S., Wilson T., Clement F. (2018). Kaplan–Meier survival analysis overestimates cumulative incidence of health-related events in competing risk settings: a meta-analysis.. J. Clin. Epidemiol..

[65] Hageman S.H.J., Dorresteijn J.A.N., Pennells L. (2023). The relevance of competing risk adjustment in cardiovascular risk prediction models for clinical practice.. Eur. J. Prev. Cardiol..

[66] Lloyd-Jones D.M., Wilson P.W.F., Larson M.G. (2004). Framingham risk score and prediction of lifetime risk for coronary heart disease.. Am. J. Cardiol..

[67] Imai Y., Mizuno Tanaka S., Satoh M. (2021). Prediction of Lifetime Risk of Cardiovascular Disease Deaths Stratified by Sex in the Japanese Population.. J. Am. Heart Assoc..

[68] Panagiotakos D., Chrysohoou C., Damigou E. (2023). Prediction of lifetime risk for cardiovascular disease, by risk factors level: the ATTICA epidemiological cohort study (2002–2022).. Ann. Epidemiol..

[69] Collett D. (2023). Modelling survival data in medical research..

[70] De Backer G., De Bacquer D. (1999). Lifetime-risk prediction: a complicated business.. Lancet.

[71] Licher S., Heshmatollah A., van der Willik K.D. (2019). Lifetime risk and multimorbidity of non-communicable diseases and disease-free life expectancy in the general population: A population-based cohort study.. PLoS Med..

[72] Bender A.P., Punyko J., Williams A.N., Bushhouse S.A. (1992). A standard person-years approach to estimating lifetime cancer risk.. Cancer Causes Control.

[73] Qwasmeh A.A.H., Saleh B.A.A. (2023). Radiation dose and lifetime risk for radiation-induced cancer due to natural radioactivity in tap water from Jordan.. Radiat. Environ. Biophys..

[74] Aldekheel M., Farahani V.J., Sioutas C. (2023). Assessing Lifetime Cancer Risk Associated with Population Exposure to PM-Bound PAHs and Carcinogenic Metals in Three Mid-Latitude Metropolitan Cities.. Toxics.

[75] Otansev P., Bingöldağ N. (2022). Indoor Radon Concentration and Excess Lifetime Cancer Risk.. Radiat. Prot. Dosimetry.

[76] Wetmore J.B., Otarola L., Paulino L.J. (2022). Estimating lifetime risk for breast cancer as a screening tool for identifying those who would benefit from additional services among women utilizing mobile mammography.. J. Cancer Policy.

[77] Fraser G.E., Shavlik D. (1997). Risk factors, lifetime risk, and age at onset of breast cancer.. Ann. Epidemiol..

[78] Bruder C., Bulliard J.L., Germann S. (2018). Estimating lifetime and 10-year risk of lung cancer.. Prev. Med. Rep..

[79] Rigel D.S., Friedman R.J., Kopf A.W. (1996). Lifetime risk for development of skin cancer in the U.S. population: Current estimate is now 1 in 5.. J. Am. Acad. Dermatol..

[80] Alharfi S., Furey N., Al-Shakhshir H., Ghannoum M., Cooper G.S. (2023). Fecal Microbiome Associated with Both Colon Adenomas and Lifetime Colorectal Cancer Risk.. Dig. Dis. Sci..

[81] Grundy A., Sandhu S., Arseneau J. (2022). Lifetime caffeine intake and the risk of epithelial ovarian cancer.. Cancer Epidemiol..

[82] Dalmartello M., Vermunt J., Negri E., Levi F., La Vecchia C. (2022). Adult lifetime body mass index trajectories and endometrial cancer risk.. BJOG.

[83] Lloyd T., Hounsome L., Mehay A., Mee S., Verne J., Cooper A. (2015). Lifetime risk of being diagnosed with, or dying from, prostate cancer by major ethnic group in England 2008–2010.. BMC Med..

[84] Rowe T.W., Katzourou I.K., Stevenson-Hoare J.O., Bracher-Smith M.R., Ivanov D.K., Escott-Price V. (2021). Machine learning for the life-time risk prediction of Alzheimer’s disease: a systematic review.. Brain Commun..

[85] Seshadri S., Drachman D.A., Lippa C.F. (1995). Apolipoprotein E epsilon 4 allele and the lifetime risk of Alzheimer’s disease. What physicians know, and what they should know.. Arch. Neurol..

[86] Lobo A., Lopez-Anton R., Santabárbara J. (2011). Incidence and lifetime risk of dementia and Alzheimer’s disease in a Southern European population.. Acta Psychiatr. Scand..

[87] Oakley Browne M.A., Elisabeth Wells J., Scott K.M., Mcgee M.A. (2006). Lifetime prevalence and projected lifetime risk of DSM-IV disorders in Te Rau Hinengaro: The New Zealand Mental Health Survey.. Aust. N. Z. J. Psychiatry.

[88] Bonnewyn A., Bruffaerts R., Vilagut G., Almansa J., Demyttenaere K. (2007). Lifetime risk and age-of-onset of mental disorders in the Belgian gen-eral population.. Soc. Psychiatry Psychiatr. Epidemiol..

[89] Crowson C.S., Matteson E.L., Myasoedova E. (2011). The lifetime risk of adult-onset rheumatoid arthritis and other inflammatory autoimmune rheumatic diseases.. Arthritis Rheum..

[90] Hess K.L., Hu X., Lansky A., Mermin J., Hall H.I. (2017). Lifetime risk of a diagnosis of HIV infection in the United States.. Ann. Epidemiol..

[91] Tuomilehto J., Bahijri S. (2016). Lifetime risk of diabetes mellitus — how high?. Nat. Rev. Endocrinol..

[92] Narayan K.M.V., Boyle J.P., Thompson T.J., Sorensen S.W., Williamson D.F. (2003). Lifetime risk for diabetes mellitus in the United States.. JAMA.

[93] McMahon G.M., Hwang S.J., Fox C.S. (2017). Residual lifetime risk of chronic kidney disease.. Nephrol. Dial. Transplant..

[94] Gaillard F., Fournier C., Legendre C. (2019). Lifetime ESKD risk stratification for living kidney donor studies.. Am. J. Transplant..

[95] Wang Z., Hoy W.E. (2014). Remaining lifetime risk for developing end stage renal disease among Australian Aboriginal people with diabetes.. Diabetes Res. Clin. Pract..

[96] Melton L.J.R.D. (1990). Lifetime risk of a hip fracture.. Am. J. Public Health.

[97] Wang Y.X.J., Griffith J.F., Blake G.M. (2023). Revision of the 1994 World Health Organization T-score definition of osteoporosis for use in older East Asian women and men to reconcile it with their lifetime risk of fragility fracture.. Skeletal Radiol..

[98] Chen V., Ning H., Allen N. (2019). Lifetime Risks for Hypertension by Contemporary Guidelines in African American and White Men and Women.. JAMA Cardiol..

[99] van Riel A.C.M.J., Blok I.M., Zwinderman A.H. (2015). Lifetime Risk of Pulmonary Hypertension for All Patients After Shunt Closure.. J. Am. Coll. Cardiol..

[100] Ligthart S., van Herpt T.T.W., Leening M.J.G. (2016). Lifetime risk of developing impaired glucose metabolism and eventual progression from prediabetes to type 2 diabetes: a prospective cohort study.. Lancet Diabetes Endocrinol..

[101] Pencina M.J., D’Agostino R.B., Larson M.G., Massaro J.M., Vasan R.S. (2009). Predicting the 30-year risk of cardiovascular disease: the framingham heart study.. Circulation.

[102] Saito I. (2021). Lifetime Risk of Coronary Heart Disease in Japan.. J. Atheroscler. Thromb..

[103] Urbut S.M., Yeung M.W., Khurshid S. (2023). MSGene: Derivation and validation of a multistate model for lifetime risk of coronary artery disease using genetic risk and the electronic health record.. medRxiv.

[104] Wang Z., Hoy W.E. (2013). Lifetime risk of developing coronary heart disease in Aboriginal Australians: a cohort study.. BMJ Open.

[105] Thomas E.A., Enduru N., Tin A. (2022). Polygenic Risk, Midlife Life’s Simple 7, and Lifetime Risk of Stroke.. J. Am. Heart Assoc..

[106] Turin T.C., Kokubo Y., Murakami Y. (2010). Lifetime risk of stroke in Japan.. Stroke.

[107] Wang Y., Liu J., Wang W. (2016). Lifetime risk of stroke in young-aged and middle-aged Chinese population.. J. Hypertens..

[108] Zhao H.L., Huang Y. (2019). Lifetime Risk of Stroke in the Global Burden of Disease Study.. N. Engl. J. Med..

[109] Brugger N., Krause R., Carlen F. (2014). Effect of lifetime endurance training on left atrial mechanical function and on the risk of atrial fibril-lation.. Int. J. Cardiol..

[110] Guo Y., Tian Y., Wang H., Si Q., Wang Y., Lip G.Y.H. (2015). Prevalence, incidence, and lifetime risk of atrial fibrillation in China: new insights into the global burden of atrial fibrillation.. Chest.

[111] Heeringa J., van der Kuip D.A.M., Hofman A. (2006). Prevalence, incidence and lifetime risk of atrial fibrillation: the Rotterdam study.. Eur. Heart J..

[112] Kheirbek R.E., Fokar A., Moore H.J., Shara N., Doukky R., Fletcher R.D. (2018). Association between lifetime risk of atrial fibrillation and mortality in the oldest old.. Clin. Cardiol..

[113] Staerk L., Wang B., Preis S.R. (2018). Lifetime risk of atrial fibrillation according to optimal, borderline, or elevated levels of risk factors: cohort study based on longitudinal data from the Framingham Heart Study.. BMJ.

[114] Jaspers N.E.M., Blaha M.J., Matsushita K. (2020). Prediction of individualized lifetime benefit from cholesterol lowering, blood pressure lower-ing, antithrombotic therapy, and smoking cessation in apparently healthy people.. Eur. Heart J..

[115] Zipkin D.A., Umscheid C.A., Keating N.L. (2014). Evidence-based risk communication: a systematic review.. Ann. Intern. Med..

[116] Pierson C.A. (2015). Understanding and communicating risk.. J. Am. Assoc. Nurse Pract..

[117] Himes D.O., Root A.E., Gammon A., Luthy K.E. (2016). Breast Cancer Risk Assessment: Calculating Lifetime Risk Using the Tyrer-Cuzick Model.. J. Nurse Pract..

[118] Gail M.H., Costantino J.P., Pee D. (2007). Projecting individualized absolute invasive breast cancer risk in African American women.. J. Natl. Cancer Inst..

[119] Matsuno R.K., Costantino J.P., Ziegler R.G. (2011). Projecting individualized absolute invasive breast cancer risk in Asian and Pacific Islander American women.. J. Natl. Cancer Inst..

[120] Gail M.H., Brinton L.A., Byar D.P. (1989). Projecting individualized probabilities of developing breast cancer for white females who are being examined annually.. J. Natl. Cancer Inst..

[121] Banegas M.P., John E.M., Slattery M.L. (2017). Projecting Individualized Absolute Invasive Breast Cancer Risk in US Hispanic Women.. J. Natl. Cancer Inst..

[122] Saadatagah S., Varughese M.G., Nambi V. (2023). Coronary Artery Disease Risk Prediction in Young Adults: How Can We Overcome the Dominant Effect of Age?. Curr. Atheroscler. Rep..

[123] Stone NJ, Robinson JG, Lichtenstein AH (2014). 2013 ACC/AHA
guideline on the treatment of blood cholesterol to reduce atherosclerotic
cardiovascular risk in adults: a report of the American
College of Cardiology/American Heart Association Task Force on
Practice Guidelines. Circulation.

[124] Piepoli M.F., Hoes A.W., Agewall S. (2016). 2016 European Guidelines on cardiovascular disease prevention in clinical practice.. Eur. Heart J..

[125] Hageman S.H.J., Kaptoge S., de Vries T.I. (2023). Prediction of individual lifetime cardiovascular risk and potential treatment benefit: develop-ment and recalibration of the LIFE-CVD2 model to four European risk regions.. Eur. J. Prev. Cardiol..

[126] (2023). LIFE-CVD: A new lifetime risk score model that estimates treatment benefit. J American College of Cardiology. https://www.acc.org/Latest-in-Cardiology/Articles/2019/06/24/14/13/LIFE-CVD-A-New-Lifetime-Risk-Score-Model-That-Estimates-Treatment-Benefit.

[127] Brotons C., Moral I., Fernández D. (2019). Estimation of Lifetime Risk of Cardiovascular Disease (IBERLIFERISK): A New Tool for Cardio-vascular Disease Prevention in Primary Care.. Rev. Esp. Cardiol. (Engl. Ed.).

[128] Brotons C., Moral-Peláez I., Vicuña J., Ameixeiras C., Fernández-Lavandera C., Sánchez-Chaparro M.Á. (2023). Update and validation of the
lifetime cardiovascular risk in Spain: IBERLIFERISK2. Clínica e
Investigación en Arteriosclerosis (English Edition).

[129] Brotons C., Calvo-Bonacho E., Moral I. (2016). Comparison of application of different methods to estimate lifetime cardiovascular risk.. Eur. J. Prev. Cardiol..

[130] Dorresteijn J.A.N., Kaasenbrood L., Cook N.R. (2016). How to translate clinical trial results into gain in healthy life expectancy for individual patients.. BMJ.

[131] Berry J.D., Dyer A., Cai X. (2012). Lifetime risks of cardiovascular disease.. N. Engl. J. Med..

[132] Hippisley-Cox J, Coupland C, Robson J, Brindle P (2010). Derivation, validation, and evaluation of a new QRISK model to estimate lifetime risk of cardiovascular disease: cohort study using QResearch database. BMJ.

[133] (2014). Joint British Societies’ consensus recommendations for the prevention of cardiovascular disease (JBS3).. Heart.

[134] (2023). National Institute for Health and Care Excellence. Recommendations | Cardiovascular disease: Risk assessment and reduction, including lipid modification | Guidance | NICE..

[135] (2023). ClinRisk Ltd. QRISK3-lifetime..

[136] Livingstone S., Morales D.R., Fleuriot J., Donnan P.T., Guthrie B. (2023). External validation of the QLifetime cardiovascular risk prediction tool: population cohort study.. BMC Cardiovasc. Disord..

[137] de Vries T.I., Jaspers N.E.M., Visseren F.L.J., Dorresteijn J.A.N. (2021). An update to the lifetime-perspective CardioVascular Disease (LIFE-CVD) model for prediction of individualized lifetime benefit from cardiovascular risk factor management in apparently healthy people.. MedRxiv.

[138] (2023). U-prevent. LIFE-CVD model..

[139] Hageman SHJ, Lu W, Kaptoge S (2022). Prediction of lifetime cardiovascular risk and individual lifetime treatment benefit in four European risk regions: geographic recalibration of the LIFE-CVD model. Eur Heart J.

[140] (2023). Iberliferisk. https://www.iberliferisk.com.

[141] Marma A.K., Berry J.D., Ning H., Persell S.D., Lloyd-Jones D.M. (2010). Distribution of 10-year and lifetime predicted risks for cardiovascular disease in US adults: findings from the National Health and Nutrition Examination Survey 2003 to 2006.. Circ. Cardiovasc. Qual. Outcomes.

[142] Berry J.D., Liu K., Folsom A.R. (2009). Prevalence and progression of subclinical atherosclerosis in younger adults with low short-term but high lifetime estimated risk for cardiovascular disease: the coronary artery risk development in young adults study and multi-ethnic study of atherosclerosis.. Circulation.

[143] Paixao A.R.M., Ayers C.R., Rohatgi A. (2014). Cardiovascular lifetime risk predicts incidence of coronary calcification in individuals with low short-term risk: the Dallas Heart Study.. J. Am. Heart Assoc..

[144] Lloyd-Jones D.M., Dyer A.R., Wang R., Daviglus M.L., Greenland P. (2007). Risk factor burden in middle age and lifetime risks for cardiovascular and non-cardiovascular death (Chicago Heart Association Detection Project in Industry).. Am. J. Cardiol..

[145] Wang Y., Liu J., Wang W. (2015). Lifetime risk for cardiovascular disease in a Chinese population: the Chinese Multi–Provincial Cohort Study.. Eur. J. Prev. Cardiol..

[146] Wilkins J.T., Ning H., Berry J., Zhao L., Dyer A.R., Lloyd-Jones D.M. (2012). Lifetime risk and years lived free of total cardiovascular disease.. JAMA.

[147] (2021). European Commission. Cardiovascular diseases prevention | Knowledge for policy..

[148] World Health Organization Cardiovascular diseases (CVDs).. https://www.who.int/news-room/fact-sheets/detail/cardiovascular-diseases-(cvds).

[149] Hayıroğlu M.İ. (2019). Telemedicine: Current Concepts and Future Perceptions.. Anatol. J. Cardiol..

[150] Tekkeşin A.İ., Hayıroğlu M.İ., Çinier G. (2021). Lifestyle intervention using mobile technology and smart devices in patients with high cardio-vascular risk: A pragmatic randomised clinical trial.. Atherosclerosis.

[151] Hayıroğlu M.İ., Çınar T., Çinier G. (2021). The effect of 1-year mean step count on the change in the atherosclerotic cardiovascular disease risk calculation in patients with high cardiovascular risk: a sub-study of the LIGHT randomized clinical trial.. Kardiol. Pol..

[152] Magnussen C., Ojeda F.M., Leong D.P. (2023). Global effect of modifiable risk factors on cardiovascular disease and mortality.. N. Engl. J. Med..

[153] Katsagoni C.N., Psarra G., Georgoulis M., Tambalis K., Panagiotakos D.B., Sidossis L.S. (2020). High and moderate adherence to Mediterranean life-style is inversely associated with overweight, general and abdominal obesity in children and adolescents: The MediLIFE-index.. Nutr. Res..

